# Tumor Necrosis Factor Alpha Blockade and Multiple Sclerosis: Exploring New Avenues

**DOI:** 10.7759/cureus.18847

**Published:** 2021-10-17

**Authors:** Maryam Zahid, Alberto Busmail, Sai Sri Penumetcha, Saher Ahluwalia, Rejja Irfan, Sawleha Arshi Khan, Sai Rohit Reddy, Maria Elisa Vasquez Lopez, Lubna Mohammed

**Affiliations:** 1 Research & Development, California Institute of Behavioral Neurosciences & Psychology, Fairfield, USA; 2 Internal Medicine, California Institute of Behavioral Neurosciences & Psychology, Fairfield, USA; 3 Internal Medicine, Chalmeda Anand Rao Institute of Medical Sciences, Karimnagar, IND; 4 Internal Medicine, Shalamar Medical & Dental College, Lahore, PAK; 5 Internal Medicine, Brooklyn Medical Services, New York, USA; 6 Research and Development, California Institute of Behavioral Neurosciences & Psychology, Fairfield, USA; 7 Research, California Institute of Behavioral Neurosciences & Psychology, Fairfield, USA; 8 Gastroenterology and Hepatology, Mayo Clinic, Rochester, USA; 9 School of Medicine, Armed Forces Medical College, Pune, IND

**Keywords:** tnf alpha receptors, tnf alpha inhibitors, multiple sclerosis, atrosab, experimental autoimmune encephalomyelitis

## Abstract

Multiple sclerosis (MS) is the most common disabling disease of the central nervous system (CNS) with a progressive neurodegenerative pattern. It is characterized by demyelination of white matter in CNS and apoptosis of oligodendrocytes. Tumor necrosis factor (TNF) alpha is a major cytokine in the pathogenesis of MS. However, the failure of TNF alpha inhibitors in preclinical and clinical trials disapproved of their use in MS patients. Nevertheless, failures and misses sometimes open avenues for new hits. In the later years, it was discovered that TNF signaling is mediated via two different receptors, TNFR1 and TNFR2, both of which have paradoxical effects. TNFR1 mediates demyelination and apoptosis, while TNFR2 promotes remyelination and neuroprotection. This explained the cause of the failure of non-selective TNF alpha-blockers in MS. It also enlightened researchers that repurposing the previously formulated non-selective TNF alpha-blockers using a receptor-selective approach could lead to discovering novel biologic agents with a broader spectrum of indications and better safety profiles. This review focuses on a novel premier TNFR1 blocker, atrosab, which has been tested in animal models of MS, experimental autoimmune encephalomyelitis (EAE), where it demonstrated a reduction in symptom severity. The early promise shown by atrosab in preclinical studies has given us hope to find another revolutionary drug for MS in the future. Clinical trials, which will finally decide whether this drug can be used as a better therapeutic agent for MS or not, are still going on, but currently, there is no approved evidence regarding efficacy of these agents in treating MS.

## Introduction and background

Multiple sclerosis (MS) is an immune-mediated demyelinating disease of white matter in the central nervous system (brain and spinal cord). Young women are affected more commonly, and it is one of the frequent causes of disability in young adults [1]. There are four identified courses of disease: relapsing-remitting MS (RRMS), primary progressive MS, secondary progressive MS, and progressive-relapsing MS. Around 85% of patients with MS have a relapsing-remitting pattern [1]. Relapsing-remitting MS (RRMS) usually occurs in individuals under the age of 40 years [1]. As the name suggests, it constitutes relapse and remission phases. During a relapse, patients may experience a complex combination of new and old symptoms lasting for at least 24 hours [1]. The relapse is then followed by a remission during which symptoms may resolve partially or entirely [1].

A definitive cure for this disease is still a challenge and focus of research for neuroscientists worldwide [2]. Treatment aims to prevent and treat acute flares as well as slow down disability accumulation [1]. Acute flares are treated with steroids [1]. Various disease-modifying drugs approved by the FDA are currently being used, including interferon beta, monoclonal antibodies (natalizumab, ocrelizumab, and alemtuzumab), fingolimod, mitoxantrone, and glatiramer [2]. These drugs aim to reduce the risk of relapses and prevent the formation of new lesions seen on MRI of the brain and spinal cord [2]. Natalizumab is considered to be the most effective drug for the prevention of relapse [2]. However, its use is limited due to the high risk of progressive multifocal leukoencephalopathy (PML) caused by the John Cunningham (JC) virus in the brain [2]. More research will find more appropriate drugs with better safety profiles and therapeutic efficacy to limit disability progression in this disease.

Tumor necrosis factor-alpha (TNF alpha) is an immunomodulatory cytokine involved in physiological and pathological processes in the CNS [3]. However, its role in the pathogenesis of MS is complex due to its contradictory effects [4]. It can exert both pro-inflammatory effects in CNS, causing secondary neuronal and axonal damage and can also exert protective effects on neurons in pathological conditions [4]. The role of TNF in the disease process of MS was further supported by evidence of elevated levels of TNF in cerebrospinal fluid (CSF) of patients of MS. The same finding was observed in animal models of MS. Moreover, central and peripheral demyelinating lesions and serum and brain plaques of MS patients also showed elevated levels of TNF [4]. All these shreds of evidence proposed that TNF plays a pivotal role in the disease process of MS, and antagonism of its effects may reduce the severity of MS symptoms.

TNF-alpha inhibitors are currently used as an efficient therapeutic choice in many autoimmune and inflammatory diseases [4]. Several TNF alpha-blockers have been approved by the FDA, including etanercept, infliximab, golimumab, certolizumab, and adalimumab [3]. However, many CNS demyelination events have been reported in patients receiving anti-TNF drugs [4-6]. As a result, the pre-existing demyelinating disease was considered a contraindication for TNF alpha-blockers. Additionally, the usage of these drugs in first-degree relatives of MS patients also became controversial due to their inherent predisposition to develop MS [7]. Meanwhile, experimental trials in mice regarding the use of TNF alpha-blockers for MS treatment were also halted because of exacerbation of symptoms rather than improvement [8]. None of these studies and trials supported the use of anti-TNF drugs in MS, so this hypothesis was rejected.

However, these failed attempts prompted researchers to enhance the scope of research in TNF signaling pathways and critically revise assumed immunopathological mechanisms [9]. Further studies revealed that TNF-alpha is a pleiotropic cytokine having two ligands, soluble TNF(solTNF), and transmembrane TN (tmTNF), that acts via two different receptors, tumor necrosis factor receptor 1 and tumor necrosis factor receptor 2 (TNFR1 and TNFR2) which have opposite effects [10,11]. TNFR1 is associated with pro-inflammatory and cell apoptosis pathways, while TNFR2 mediates immune modulation, tissue regeneration, and neuroprotection [10,11]. The loss of anti-inflammatory effects mediated by TNFR2 may explain the failure of non-selective TNF blockers in treating MS in previous experimental trials [12]. This led to the idea that selective inhibition of TNFR1 can be a more precise interventional approach for treating MS patients than non-selective TNF alpha-blockers [13].

Atrosab is a humanized monovalent antibody against TNFR1 developed for the treatment of inflammatory disorders. In vitro, atrosab successfully inhibited TNF-mediated responses such as apoptosis and nuclear factor (NF) kappa B-dependent gene expression that is responsible for the production of interleukin 6 (IL-6) and interleukin 8 (IL-8) [14]. These findings paved the way to take these experimental trials to the next level, where the therapeutic potential of atrosab was tested in non-human primates [14]. Chimeric human/mice with experimental autoimmune encephalomyelitis (EAE) when treated with atrosab also showed marked improvement in symptoms and reduced disease severity [15]. The new hypothesis supported by various studies suggests that anti-TNFR1 therapy does not cause any damage to the activity of immune cells or their composition; instead, it restricts the penetration of immune cells across the blood-brain barrier by inhibiting TNF-induced adhesion molecules [15]. Keeping in view the mechanism of TNF signaling and the successful preclinical trials, we hope that the selective TNFR1 blocker (atrosab) can be used as a novel therapy in MS and other TNF-mediated diseases. This drug can also be a better alternative in diseases where anti-TNF drugs are already being used [16].

We searched the PubMed database using the Medical Subject Headings (MeSH) keywords: receptors, tumor necrosis factor, type 1. We conducted additional data search on PubMed using regular keywords: TNF alpha selective inhibitors in multiple sclerosis and atrosab. In this review, we included all the studies related to the pathophysiological mechanisms of TNF signaling in neurodegenerative diseases, the use of TNF alpha-blockers in other autoimmune diseases, the adverse effects, and the efficacy of TNF alpha-blockers.

## Review

Role of tumor necrosis factor and its receptors in neurodegenerative diseases

TNF is a regulatory cytokine and is a critical signaling protein involved in the pathophysiology of various immune-mediated and inflammatory disorders, including neurodegenerative diseases [17]. Williams et al. showed that elevated levels of TNF are found in the serum and CSF of patients with MS, and it shows a positive correlation with symptom severity [8]. Kollias and Kontoyiannis concluded from the results of murine disease models that TNF alpha receptor exerts its effects via two receptors, TNFR1 and TNFR2, both carrying contradictory effects [18]. They also suggested that drugs targeting TNF alpha receptors may provide a better therapeutic option for autoimmune diseases over non-selective TNF alpha inhibitors [18]. Taoufik et al. (2011) presented the evidence that TNF exists in two forms, soluble and transmembrane, which have opposing effects in MS [19]. Their study showed that the soluble form of TNF promotes inflammation via TNFR1 in the CNS, whereas the transmembrane form provides neuroprotection via the TNFR2 signaling pathway [19]. Doss et al. discovered that receptors of the TNF family stimulate two opposite signaling pathways: TNFR1 stimulating apoptosis pathways and TNFR2 stimulating cell survival pathways [20]. Puimège et al. found that TNFR1 is ubiquitously expressed on most cells, while TNFR2 has a limited expression which is immunoregulatory [17]. Madsen et al. also presented similar findings, “multiple sclerosis is associated with pro-inflammatory effects of solTNF mediated through TNFR1,” while TNFR2 promotes repair and remyelination [21]. This study was the first to provide direct evidence that TNFR2 is required for the repair and differentiation of oligodendrocytes by using novel TNFR2 knockout (KO) mice and selective TNFR2 ablation in oligodendrocytes [21]. Dong et al. successfully demonstrated the neuroprotective role of TNFR2 by selectively inhibiting TNFR1 using atrosab (TNFR1 selective antagonistic antibody) and by activating TNFR2 using EHD2-scTNFR2 (a TNFR2 selective agonist) in a mouse model of N-methyl-D-aspartate (NMDA)-induced neurodegeneration [11]. Gane et al. explained autocrine feedback loops of TNF alpha in human monocytes [22]. Autocrine binding of TNF alpha led to upregulation of pro-inflammatory cytokines via TNFR1, while autocrine binding with TNFR2 led to the production of the anti-inflammatory cytokine interleukin-10 (IL-10) [22]. Medler and Wajant also emphasized the protective and anti-inflammatory effects of TNFR2 in oligodendrocytes, cardiomyocytes, and keratinocytes [12].

Although TNF is a precise therapeutic target for the treatment of MS, a complete blockade of TNF alpha effects may not yield desired outcomes due to the heterogeneity of receptors. A better therapeutic strategy may be designed by being specific for either of the two anti pathways; antagonism of TNFR1 or agonizing the effects of TNFR2. Table 1 gives a brief summary regarding the dualistic role of TNF alpha in neurodegenerative diseases.

**Table 1:** Literature summary illustrating role of TNF alpha in neurodegenerative diseases TNF: tumor necrosis factor; TNFR: tumor necrosis factor receptor; MS: multiple sclerosis; EAE: experimental autoimmune encephalomyelitis; NMDA: N-Methyl-D-aspartate; IL: interleukin

Study	Chronology	Study Outcome
Taoufik et al. [19]	2011	This study successfully demonstrated divergent effects of soluble and transmembrane forms of TNF in a mouse model of MS and supported the concept of selective inhibition of TNFR1.
Doss et al. [20]	2014	This study supports the opposing effects of TNF alpha on its receptors, TNFR1 and TNFR2, and their implication as a drug target for autoimmune disorders.
Puimège et al. [17]	2014	This study found pro-inflammatory effects of TNF and its receptors. TNFR1 is predominant and pro-inflammatory, and TNFR2 mediates immune regulatory effects.
Madsen et al. [21]	2016	This study gave the first direct evidence that TNFR2 is required for oligodendrocyte differentiation in mice model of MS.
Dong et al [11]	2016	In this study, the therapeutic potential of selective inhibition of TNFR1 and activation of TNFR2 was successfully demonstrated in a mouse model of NMDA-induced neurodegeneration. However, complete blocking of TNF effects by non-selective inhibitors diminished the therapeutic effects.
Gane et al. [22]	2016	This study led to the conclusion that in monocytes, TNFR1 caused upregulation of pro-inflammatory cytokines, while TNFR2 caused an increase in anti-inflammatory cytokines such as IL-10. Moreover, the TNFR1 blockade did not affect the expression of TNFR2.
Medler and Wajant [7]	2019	Targeting of TNFR2 may be used to treat cancer and autoimmune diseases due to its protective and anti-inflammatory effects on cardiomyocytes, keratinocytes, and oligodendrocytes.

TNF-alpha inhibitors are remarkable in treating inflammatory bowel disease and rheumatological conditions including rheumatoid arthritis and ankylosing spondylitis [23]. Other indications for using these drugs are psoriasis, asthma, uveitis, and vasculitis, etc. [23]. These drugs have shown considerable benefits in reducing chronic damage in Crohn’s disease and rheumatoid arthritis, which has led investigators to propose using these drugs early in the course of the disease to control the symptoms quickly [24]. Due to the widespread usage of TNF alpha-blockers, there is now a better understanding of the adverse effects and long-term tolerability of these drugs [24]. Demyelinating neurological damage is a rare but potentially lethal side effect seen after the onset of therapy in previously healthy subjects, as confirmed by scattered case reports from various countries and by systematic reviews [5]. Lin et al. stated that a demyelinating neurological disorder similar to MS is a continuously reported side effect seen with anti-TNF therapy [25]. Piusińska-Macoch showed case reports of various neurological adverse events seen with the use of TNF alpha-blockers which include serious viral infections such as herpes simplex virus (HSV) and JC virus, and neurological bacterial infections such as *Listeria monocytogenes* [23]. Several cases of PML and herpes simplex encephalitis were seen in the first six months of using these drugs [23]. Other cases were of central and peripheral demyelination disorders similar to MS, optic neuropathy, and Guillain-Barre syndrome (GBS) [23]. Fernández-Espartero et al. conducted a systematic review to estimate demyelinating disease rates in patients treated with TNF alpha-blockers [26]. They could not demonstrate whether demyelination events were due to TNF alpha exposure or due to some other reason [26]. Mansouri et al. argued against the use of TNF alpha-blockers in people at risk of developing MS, that is, first-degree relatives of MS patients [7]. The data presented by their study concluded that the number needed to treat was relatively less than the number needed to harm [7]. They suggested that physicians should weigh the risks versus benefits and work with neurologists while prescribing TNF alpha-blockers to this subset of patients, rather than completely restricting their use in the first-degree relatives of MS patients [7]. Cohen et al. also assessed the frequency of neurological demyelination symptoms after receiving anti-TNF therapy [27]. They also found an association, and they suggested that Tintore criteria and CSF analysis can be helpful in clinical practice to diagnose a first demyelinating event [27]. Singh et al. also demonstrated that anti-TNF therapy could stimulate a neurological disease similar to MS [28]. Napolitano et al. presented a case report of MS in a 48-year-old man with plaque-psoriasis treated with etanercept [29]. The literature review highlighted 35 cases that developed demyelinating diseases during treatment [29]. Zhu et al. conducted a review to assess the demyelination risk in psoriasis patients receiving anti-TNF therapy [30]. They concluded that although the association between anti-TNF therapy and demyelinating effects exists, the incidence is very low [30]. Kopp conducted the most recent cohort study to assess the risk of developing neurodegenerative diseases in arthritis patients treated with anti-TNF drugs [31]. They concluded that the use of TNF alpha-blockers in ankylosing spondylitis and psoriatic arthritis, not in rheumatoid arthritis, was associated with developing demyelinating disorders [31]. The absolute risk was very low, about 1 in 1000 cases per year [31].

We can deduce from the above studies that although adverse neurological outcomes rarely occur with the use of TNF alpha-blockers, physicians should still screen patients for risk factors before starting treatment so that iatrogenic harm is minimized. Most susceptible individuals are those who have a first-degree relative with MS. Moreover, if any neurological symptoms appear during treatment, the drug should be immediately discontinued, and proper evaluation of the patient should be done because the ultimate goal of using these biological agents is to benefit the patient and not to provide cure at the cost of other illnesses.

Selective inhibition of TNFR1 improved MS symptoms

 A more targeted and selective approach toward TNF receptors might prove more beneficial in the treatment of MS than non-selective treatment. Experimental trials were redesigned to selectively inhibit TNFR1 to control pro-inflammatory effects or activate TNFR2 to promote anti-inflammatory and cell survival pathways.

**Figure 1:**
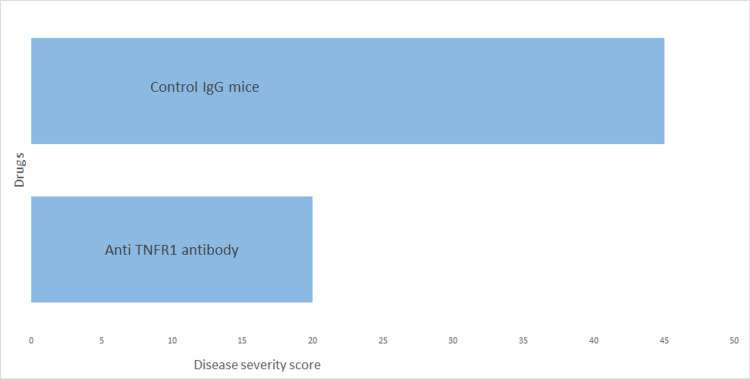
Experimental trials in EAE mice show a decrease in symptoms severity after treatment with anti-TNFR1 antibody IgG: immunoglobulin G; TNFR: tumor necrosis factor receptor; EAE: experimental autoimmune encephalomyelitis

Caminero et al. hypothesized in their study that selective TNFR1 inhibition may lead to more favorable results in MS [32]. Van Hauwermeiren et al. also supported the hypothesis of selectively inhibiting TNFR1 to achieve better therapeutic outcomes in MS and other diseases where TNFR2 blockade may be harmful [33]. Nomura et al. claimed that PEGylated anti-TNFR1 blocker showed remarkable efficacy in reducing symptoms of cerebral demyelination in EAE [34]. Williams et al. also conducted experimental trials to confirm the efficacy of TNFR1 selective antibody in mice with EAE. They found a marked improvement in symptoms following both prophylactic and therapeutic regimes [8]. These preclinical trials demonstrated that TNFR1-deficient mice had showed reduced incidence and severity of disease than wild-type (WT) mice, while TNFR2-deficient mice showed an increased severity of symptoms [8]. Histopathological analysis of the spinal cord also showed markedly reduced demyelination compared to control animals [8]. Gane et al. also supported the concept that selective TNFR1 blockade can give beneficial results while preserving protective effects mediated by TNFR2 [23]. Dong et al. conducted a study in which they selectively targeted TNF receptors in a mouse model of NMDA-induced neurodegeneration [11]. They concluded that inhibiting TNFR1 and stimulating TNFR2 protected cholinergic neurons from apoptosis and reversed some of the neurological impairments. However, complete blockade did not produce any therapeutic benefits [11]. Yang et al. also proposed that more appropriate novel drugs can be formulated by either antagonizing TNFR1 effects or using TNFR2 agonists, yielding promising results by promoting tissue regeneration and remyelination [10]. Williams et al. also corroborated the therapeutic efficacy of using anti-human TNFR1 drugs in autoimmune neurodegenerative diseases [15]. Medler and Wajant argued in favor of using TNFR2 as a drug target for neurodegenerative disease due to its established role in promoting remyelination and cell regeneration [12]. Richter et al. also favored selective TNFR1 inhibition as a superior choice than complete TNF alpha-blockers as the former retains the protective effects mediated by TNFR2 [13]. Ribeiro et al. also found that increased TNFR1 was positively associated with progressive MS and increased TNFR2 was negatively associated with progressive MS [35]. Figure 1 describes how anti-TNFR1 antibody has shown promise in preclinical trials. We can see that mice treated with anti-TNFR1 antibody showed marked reduction in symptom severity as compared to those treated with control IgG.

The above studies enlighten us that we can widen the therapeutic spectrum of these drugs by selectively targeting the TNF alpha receptors while ameliorating the neurological adverse effects reported with non-selective TNF alpha blockade. The success of selective TNFR1 blockers in preclinical trials further verified this hypothesis. These drugs can provide better treatment options in MS and other diseases where complete blockade of TNF effects was not effective. In addition, it can also be used as a revised strategy in other TNF-mediated diseases, that is, rheumatoid arthritis, inflammatory bowel disease, and psoriasis.

Atrosab, a novel biological drug with anti-TNFR1 activity

**Figure 2:**
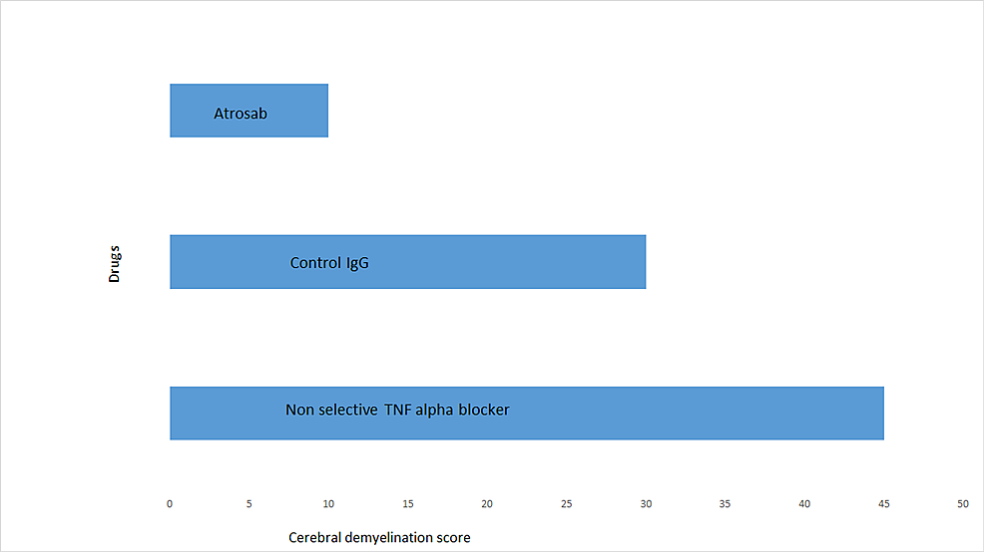
Treatment with atrosab improves cerebral demyelination in EAE in contrast to non-selective TNF alpha-blocker which exacerbated the disease EAE: experimental auto-immune encephalomyelitis; TNF: tumor necrosis factor; IgG: immunoglobulin G

Understanding the effects of selective TNF receptors blockade and its success in preclinical trials led to the formulation of a novel biological agent with anti-TNFR1 activity, which can be tested and approved for the treatment of TNF-mediated diseases, especially those in which non-selective TNF alpha-blockers were proven to be detrimental [36]. Kontermann et al. described humanization and functional properties of TNFR1 antagonist antibody fragment that only blocked proapoptotic effects mediated by TNFR1, but the TNFR2 pathway remained unaffected [16]. Nomura et al. also conducted a study to increase the TNFR1 selectivity and antagonistic activity of previously developed anti-TNFR1 selective mutant (mutTNF) by phage display technology [37]. Out of 20 variants isolated, one showed 40 times more selectivity for TNFR1 than mutTNF [37]. Zettlitz et al. described successful inhibition of TNFR1-mediated effects in vitro by a mouse anti-human TNFR1 monoclonal antibody that was humanized and converted into an immunoglobulin G (IgG1) molecule (atrosab) [14]. Richter et al. also concluded in their study that atrosab can be used as an effective TNFR1 antagonist [38]. Richter et al. reengineered variable domains of atrosab to generate a monovalent Fab derivative of atrosab with increased affinity and inhibitory potential for TNFR1 [39]. Richter et al. further improved the pharmacokinetics of previously generated anti-TNFR1 monoclonal antibody fragment by heterodimerization technology [13]. They named the resultant fusion protein Atrosimab [13]. Saddala and Huang (2019) recently discovered novel TNF alpha inhibitors, TNFR1 and TNF alpha-TNFR1 complex using zinc database [36]. These compounds were found to have no toxicity and are considered promising anti-inflammatory candidates to be explored further for clinical efficacy [36]. Figure 2 illustrates how atrosab has shown its therapeutic efficacy in animal models of MS by reducing the cerebral demyelination score in contrast to non-selective TNF alpha blocker which caused further deterioration.

Thus we can see that after identifying the selective TNF receptor blockers, much work has been done to modify these biological molecules to increase their efficacy and safety so that they can be considered clinically appropriate therapeutic choices. Although atrosab has shown promise in preclinical trials, it has yet to pass clinical trials in human subjects before being recruited as an approved treatment option for MS and other TNF-mediated autoimmune disorders.

These reformulated drugs can also be tested for use in other diseases such as psoriasis, inflammatory bowel disease, rheumatoid arthritis, and ankylosing spondylitis, where non-selective TNF alpha-blockers have been approved but their use is limited due to demyelinating side effects.

Limitations

This study is a traditional literature review, and the articles reviewed have not undergone any quality appraisal. Most of the studies included in this review are animal studies. Data search was not conducted systematically. Currently, we have no data of any clinical trials that depict how beneficial atrosab has been so far in reducing disability progression in patients with MS.

## Conclusions

The idea of receptor-selective modulation of TNF alpha appears promising based on the information currently available. While acknowledging the benefits of non-selective TNF alpha-blockers already in the market, we can apprehend that selective receptor blockade can be more rewarding in terms of therapeutic value and safety profile. The recent promise shown by atrosab, a premier selective TNFR1 blocker, gives us hope to discover a better drug for MS. We can also anticipate that this repurposing approach will open new horizons to discover more innovative drugs for many other diseases. Clinical trials are being conducted to validate the usage of selective TNFR1 blockers in human subjects. They may be a revolutionary advancement in the field of immunobiology if they demonstrate similar outcomes shown in preclinical trials. More studies should also be done by conducting extensive phase 3 and phase 4 clinical trials to evaluate the effectiveness of selective TNF alpha-blockers.
